# Clinical trajectories of critically ill patients discharged directly from a critical unit to a postacute care facility: retrospective cohort

**DOI:** 10.62675/2965-2774.20240015-en

**Published:** 2024-08-07

**Authors:** João Gabriel Rosa Ramos, Milton José de Souza, Alef Santiago Rezende, Flavia dos Santos Ferreira, Yanne Danielly Santos Amorim, Flaviane Ribeiro de Souza, Lucas Freire de Andrade

**Affiliations:** 1 Clínica Florence Salvador BA Brazil Clínica Florence - Salvador (BA), Brazil.; 2 Universidade Federal da Bahia Faculdade de Medicina Salvador BA Brazil Faculdade de Medicina, Universidade Federal da Bahia - Salvador (BA), Brazil.

**Keywords:** Patient discharge, Functional status, Length of stay, Hospitalization, Subacute care, Delivery of health care, Palliative care, Aged, Intensive care units

## Abstract

**Objective:**

To describe the clinical trajectories of patients discharged directly from a critical unit to a postacute care facility.

**Methods:**

This was a retrospective cohort study of patients who were transferred from an intensive care unit or intermediate care unit to a postacute care facility between July 2017 and April 2023. Functional status was measured by the Functional Independence Measure score.

**Results:**

A total of 847 patients were included in the study, and the mean age was 71 years. A total of 692 (82%) patients were admitted for rehabilitation, while 155 (18%) were admitted for palliative care. The mean length of stay in the postacute care facility was 36 days; 389 (45.9%) patients were discharged home, 173 (20.4%) were transferred to an acute hospital, and 285 (33.6%) died during hospitalization, of whom 263 (92%) had a do-not-resuscitate order. Of the patients admitted for rehabilitation purposes, 61 (9.4%) had a worsened functional status, 179 (27.6%) had no change in functional status, and 469 (63%) had an improved functional status during hospitalization. Moreover, 234 (33.8%) patients modified their care goals to palliative care, most of whom were in the group that did not improve functional status. Patients whose functional status improved during hospitalization were younger, had fewer comorbidities, had fewer previous hospitalizations, had lower rates of enteral feeding and tracheostomy, had higher Functional Independence Measure scores at admission to the postacute care facility and were more likely to be discharged home with less complex health care assistance.

**Conclusion:**

Postacute care facilities may play a role in the care of patients after discharge from intensive care units, both for those receiving rehabilitation and palliative care, especially for those with more severe illnesses who may not be discharged directly home.

## INTRODUCTION

Intensive care unit (ICU) survivors may have impaired quality of life and worse long-term outcomes.^( 1 )^ As such, models of care for patients post-ICU discharge, such as post-ICU clinics, have been proposed.^( 2 , 3 )^ However, some of these patients have prolonged ICU stays, with more severe and protracted illness, and may not be directly discharged home after clinical stabilization.^( 4 )^ The most appropriate model of care for these patients is not well established,^( 5 )^ but the utilization of postacute care facilities (PACFs) is an alternative model, with up to 30% of acutely ill patients in the United States being discharged from an acute care facility to a PACF.^( 6 )^ In this study, we collected data from patients discharged directly from an ICU or intermediate care unit (IMCU) to a PACF in Salvador, Brazil.

## METHODS

This retrospective cohort study was approved by the Ethics Committee with a waiver for consent (approval 53032821.0.0000.0047). All patients who were transferred directly from an ICU or IMCU to the PACF between July 2017 and April 2023 were included in the study. The PACF is a private 60-bed facility with 24/7 nursing and physician care. The PACF allows patients to be admitted for rehabilitation or palliative care, including patients who require mechanical ventilation and renal replacement therapy. Unstable patients, such as those requiring vasoactive drugs, are not suitable for admission. Rehabilitation services, including physical therapy, speech and language therapy and occupational therapy, are provided daily. Patients referred for rehabilitation may receive up to 18 hours of therapy per week in the first week of hospitalization. Other clinicians, such as social workers, dietitians, psychologists, and clinical pharmacists, are also part of the team in this facility.

Functional status was measured weekly by the Functional Independence Measure (FIM) (scores ranging from 18 to 126 points). Patients were categorized as having total functional dependence (FIM score = 18), severe functional dependence (FIM score between 19 and 60 points), moderate functional dependence (FIM score between 61 and 103 points) or slight functional dependence/independence (FIM score between 104 and 126 points). Patients were also categorized as having a worsened functional status, no change in functional status or improved functional status, according to the difference between the last measured and admission FIM score.

Differences between groups of patients were assessed by ANOVA or chi-square tests for continuous or categorical variables, respectively. Post hoc Bonferroni correction was used to assess differences between each pair of categories. Repeated measures of FIM scores were evaluated by paired t tests or McNemar tests for continuous or categorical variables, respectively. A value of p < 0.05 was considered statistically significant. All analyses were performed with Statistical Package for the Social Sciences (SPSS) v 21.0.

## RESULTS

During the study period, there were 2,022 patients admitted to the PACF from 17 different hospitals, of whom 847 (42%) were admitted directly from the ICU or IMCU and were included in the study. Most patients (692 (82%)) were admitted for rehabilitation, while 155 (18%) were admitted for palliative care.

The patients were elderly, with a mean age ± standard deviation (SD) of 71 ± 17 years, a mean ± SD of 2.6 ± 1.8 comorbidities and a mean ± SD length of stay in the acute hospital of 35 ± 31 days ( Table 1 ). The most frequent reasons for admission to the acute hospital were infection with coronavirus disease 2019 (COVID-19) (i.e., patients with a positive severe acute respiratory syndrome coronavirus 2 [SARS-CoV-2] test and symptoms compatible with COVID-19), which was present in 164 (25%) patients, and sepsis, stroke, malignancies and trauma or urgent surgery, which were present in 123 (18%), 116 (17%), 71 (11%) and 66 (1%) patients, respectively. Regarding discharge disposition, 389 (45.9%) patients were discharged home, 173 (20.4%) patients were transferred to an acute hospital, and 285 (33.6%) patients died, of whom 263 (92%) had a do-not-resuscitate order. The median (interquartile range - IQR) length of stay was 30 (12 - 52) days for all patients and 45 (29 - 63), 16 (7 - 32) and 16 (7 - 35) days for patients who were discharged home, patients who died and patients who were transferred to an acute hospital, respectively.

**Table 1:** Characteristics of patients admitted to the postacute care facility directly from an intensive care unit or intermediate care unit (n = 847), patients referred for palliative care and rehabilitation, and patients referred for rehabilitation with different functional trajectories (n = 649)

Characteristics		All patients (n = 847)	Referred for palliative care (n = 155)	Referred for rehabilitation (n = 692)	p value *	Referred for rehabilitation with at least 2 FIM score measurements during PACF hospitalization (n = 649)			
						Worsened functional status during hospitalization (n = 61)	No change in functional status during hospitalization (n = 179)	Improved functional status during hospitalization (n = 409)	p value †
Male sex		441 (52.1)	76 (49)	365 (52.7)	0.403	27 (44.3)	99 (55.3)	215 (52.6)	0.329
Age		71 ± 17	80 ± 15	69 ± 17	< 0.001	77 ± 14	71 ± 18	67 ± 17	< 0.001 ‡ § ¶
Number of comorbidities		2.6 ± 1.8	2.1 ± 1.9	2.6 ± 1.8	0.006	3.2 ± 1.8	2.6 ± 1.9	2.6 ± 1.8	0.042 §
Hospitalizations in the previous year		0.86 ± 1.2	1.27 ± 1.48	0.76 ± 1.11	< 0.001	1.11 ± 1.26	0.81 ± 1.13	0.69 ± 1.05	0.017 §
Length of stay in the acute hospital (days)		35 ± 31	24 ± 23	38 ± 32	< 0.001	36 ± 33	44 ± 38	36 ± 29	0.012 ¶
Unit before transfer to the PACF					0.022				0.703
	ICU	543 (64.1)	87 (56.1)	456 (65.9)		38 (62.3)	122 (68.2)	273 (66.7)	
	IMCU	304 (35.9)	68 (43.9)	236 (34.1)		23 (37.7)	57 (31.8)	136 (33.3)	
Enteral feeding at admission to the PACF		567 (66.9)	97 (62.6)	470 (67.9)	0.202	43 (70.5)	153 (85.5)	2487 (60.4)	< 0.001 ‡ ¶
Tracheostomy at admission to the PACF		288 (34)	28 (18.1)	260 (37.6)	< 0.001	22 (36.1)	98 (54.7)	129 (31.5)	< 0.001 ‡ ¶
Mechanical ventilation at admission to the PACF		35 (4.1)	8 (5.2)	27 (3.9)	0.476	3 (4.9)	9 (5)	13 (3.2)	0.508
FIM at admission to the PACF		3 5 ± 19	27 ± 20	37 ± 19	< 0.001	35,7 ± 15,9	26,4 ± 16,6	41,4 ± 19,2	< 0.001 ‡ ¶
Last measured FIM at PACF hospitalization, mean (SD)		48 ± 32	27 ± 20	51 ± 32	< 0.001	26,7 ± 13,5	26,4 ± 16,6	66,6 ± 31,1	< 0.001 § ¶
FIM categories at admission					< 0.001				< 0.001 ‡ ¶
	Total functional dependence	191 (24.7)	59 (54.6)	132 (19.8)		0 (0)	99 (55.3)	31 (7.6)	
	Severe functional dependence	501 (64.8)	42 (38.9)	459 (69.0)		55 (90.2)	73 (40.8)	319 (78.0)	
	Moderate functional dependence	72 (9.3)	5 (4.6)	67 (10.1)		6 (9.8)	5 (2.8)	54 (13.2)	
	Slight functional dependence/independence	9 (1.2)	2 (1.9)	7 (1.1)		0 (0)	2 (1.1)	5 (1.2)	
FIM categories at last measurement during PACF hospitalization					< 0.001				< 0.001 § ¶
	Total functional dependence	187 (24.5)	61 (59.2)	126 (19.1)		25 (41.0)	99 (55.3)	0 (0)	
	Severe functional dependence	335 (43.9)	33 (32.0)	302 (45.8)		34 (55.7)	73 (40.8)	190 (46.5)	
	Moderate functional dependence	168 (22.0)	7 (6.8)	161 (24.4)		2 (3.3)	5 (2.8)	150 (367)	
	Slight functional dependence/independence	73 (9.6)	2 (1.9)	71 (10.8)		0 (0)	2 (1.1)	69 (16.9)	
Changes of goals-of-care to palliative care		234 (27.6)	NA	234 (33.8)	NA	40 (65.6)	93 (52)	83 (20.3)	< 0.001 § ¶
Length of stay in the PACF)		36 ± 34	22.6 ± 25	39.2 ± 35	< 0.001	41 ± 29	33 ± 51	44 ± 25	0.002 ¶
Discharge disposition					< 0.001				< 0.001 ‡ § ¶
	Death	285 (33.6)	127 (81.9)	169 (24.4)		31 (50.8)	86 (48)	27 (6.6)	
	Transference to acute hospital	173 (20.4)	4 (2.6)	158 (22.8)		11 (18)	59 (33)	81 (19.8)	
	Discharged home	389 (45.9)	24 (15.5)	365 (52.7)		19 (31.1)	34 (19)	301 (73.6)	
Death with a do-not-resuscitate order among deceased patients		263 (92.2)	127 (100)	136 (80.4)	< 0.001	30 (96.8)	71 (82.5)	21 (77.8)	< 0.001 ‡ §
Complexity of health care assistance after discharge from the PACF					0.196				< 0.001 § ¶
	Discharged home without home assistance	104 (12)	4 (3)	100 (14)		1 (2)	5 (3)	93 (23)	
	Discharged home with home-based rehabilitation services	232 (27)	14 (9)	218 (32)		9 (15)	8 (4)	191 (47)	
	Discharged home with 24-hour nursing care	54 (6)	6 (4)	48 (7)		9 (15)	21 (12)	17 (4)	
Satisfaction score with hospitalization in the PACF		9.5 ± 1	9.5 ± 1	9.5 ±1	0.681	9.4 ± 0.9	9.5 ± 1.1	9.5 ± 1.1	0.718

FIM - Functional Independence Measure; PACF - postacute care facility; ICU - intensive care unit; IMCU - intermediate care unit.

* p value for comparison between patients admitted for palliative care or rehabilitation;

† p value for comparison between patients with worsened functional status, no change in functional status and improved functional status during hospitalization;

‡ p < 0.05 between worsened functional status during hospitalization *versus* no change in functional status during hospitalization;

§ p < 0.05 between worsened functional status during hospitalization *versus* improved functional status during hospitalization;

¶ p < 0.05 between no change in functional status during hospitalization *versus* improved functional status during hospitalization. Results expressed as n (%) or mean ± standard deviation.

Patients admitted for palliative care were older, had more comorbidities, had more previous hospitalizations and presented with more severe functional dependence at admission to the PACF ( Table 1 ) than patients admitted for rehabilitation. Nevertheless, palliative care patients had a shorter length of stay in the acute hospital and lower rates of tracheostomy. Patients referred for palliative care stayed in the PACF for shorter periods were more likely to die than patients referred for rehabilitation.

Of the patients admitted for rehabilitation purposes, 649 (93.8%) had at least two FIM score measurements. A comparison of the last measured FIM score with the admission FIM score revealed that 61 (9.4%) patients had a worsened functional status, 179 (27.6%) had no change in functional status, and 469 (63%) had an improved functional status during hospitalization. Patients whose functional status improved during hospitalization were younger, had fewer comorbidities, had fewer previous hospitalizations, had lower rates of enteral feeding and tracheostomy, and had higher FIM scores at admission to the PACF ( Table 1 ). Patients with improved functional status were less likely to modify their goals-of-care to palliative care, had a shorter length of stay in the PACF and were more likely to be discharged home with less complex health care assistance.

**Figure 1:**
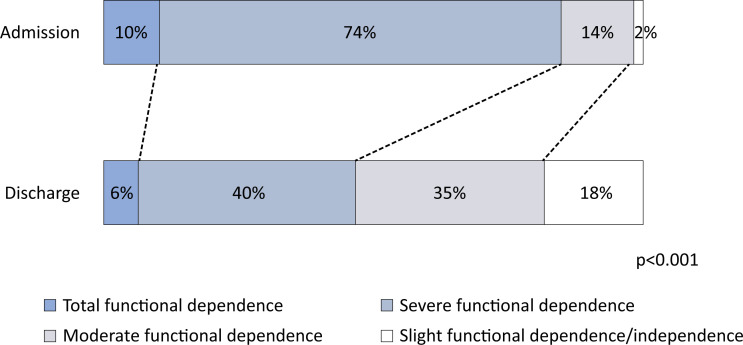
Changes in functional categories during hospitalization for patients admitted for rehabilitation, with at least two Functional Independence Measure score measurements, who were discharged home, n = 354 (p < 0.001).

For patients who were admitted for rehabilitation purposes and were discharged home and had at least two FIM score measurements (n = 354), there was an improvement in the mean FIM score, from a mean ± SD score of 41.3 points ± 20.4 at admission to a mean ± SD score of 66.1 points ± 33.3 at discharge (p < 0.001), with the functional status of 301 (85%) patients improving during hospitalization. This improvement was also observed in the analysis of the categorized FIM scores. Comparing admission scores to discharge scores, there was a reduction in the proportion of patients with total functional dependence (from 37 (10%) to 23 (6%)) and with severe functional dependence (from 263 (74%) to 142 (40%)), and an increase in the proportion of patients with moderate functional dependence, from 48 (14%) to 124 (35%), and of slight functional dependence/independence, from 6 (2%) to 65 (18%), p < 0.001, with 175 (49.4%) patients improving functional status category during hospitalization ( Figure 1 ).

## DISCUSSION

In this study, we reported that patients who were transferred from ICUs or IMCUs to the PACF were elderly patients with multimorbidity, prolonged ICU stays and increased clinical complexity. Most patients were admitted for rehabilitation, and it was possible to identify three different trajectories of functional status, along with variables that were associated with each trajectory. Moreover, the modification of goals of care to palliative care was frequently observed in this population.

The severity of functional dependence in our study population indicates lower access to conventional follow-up clinics, reinforcing the need for specific research in alternative models of care.^( 5 , 7 )^ However, most studies in post-ICU care facilities have focused on a broad population of less severely impaired patients. For instance, patients included in a multicomponent sepsis transition trial were younger and had lower rates of ICU admission and a shorter length of stay in the acute hospital.^( 8 , 9 )^

The rates of return to acute hospitals were comparable to those in the literature^( 10 )^ and were not different from the rates of rehospitalization in general post-ICU and sepsis patients.^( 8 , 9 , 11 )^ However, the overall mortality rate was greater than that previously reported for general patients post-ICU admission.^( 12 )^ Nevertheless, the greater severity of functional dependence in this population indicates the possibility of selection bias in the mortality and rehospitalization analyses because previous studies usually followed long-term outcomes after discharge home, not including deaths that occur in PACFs or that would occur in the acute hospital in the absence of transfer to a PACF.

We were able to evaluate differences in the characteristics of patients transferred for palliative care and for rehabilitation, and we found that most patients admitted for rehabilitation had significant functional gains. Additionally, we were able to describe three different groups of patients admitted for rehabilitation, with different functional trajectories during hospitalization that were associated with clinical characteristics and outcomes. These findings may help improve patient prognosis and align the expectations of patients and their relatives.

Approximately 30% of the patients modified their goals of care during hospitalization. Fewer than half of patients usually have treatment plans that are formally aligned with their preferences,^( 8 , 9 )^ even though this is one of the proposed post-ICU care elements.^( 13 )^ We do not have the exact timing of the modification of goals of care, so it was not possible to ascertain the direction of the association between the modification of goals of care and the clinical trajectory during hospitalization. However, given the primary objective of rehabilitation and the greater proportion of patients with care goal modifications among patients whose functional status did not improve during hospitalization, we hypothesize that the modification of treatment plans was influenced by a lack of response to the therapies provided.

This was an observational, retrospective study, so our findings should be considered hypothesis-generating. Moreover, despite including patients referred from 17 different hospitals, this was a single-center study with limited access to acute hospitalization data. Nevertheless, to our knowledge, this is the first study to address critical admissions to a PACF in Brazil, and our results may help inform patients, clinicians and policy-makers on the utilization of PACFs as an alternative discharge location for critically ill patients.

## CONCLUSION

Postacute care facilities may play a role in the care of patients after intensive care unit admission, especially for those with more severe illnesses who may not be discharged directly home.
