# Supplementary material for: Clinical trajectories of critically ill patients discharged directly from a critical unit to a postacute care facility: retrospective cohort
Source: Crit Care Sci. 2024 Aug 7;36:e20240015en. doi: 10.62675/2965-2774.20240015-en (PMC11463978; doi:10.62675/2965-2774.20240015-en)
Supplement: Translation

# Translation: Trajetórias clínicas de pacientes graves que recebem alta diretamente de uma unidade de terapia intensiva para uma unidade de cuidados pós-agudos: uma coorte retrospectiva

**DOI:** 10.62675/2965-2774.20240015-pt

**Authors:** João Gabriel Rosa Ramos, Milton José de Souza, Alef Santiago Rezende, Flavia dos Santos Ferreira, Yanne Danielly Santos Amorim, Flaviane Ribeiro de Souza, Lucas Freire de Andrade

**Affiliations:** 1 Salvador BA Brasil Clínica Florence - Salvador (BA), Brasil.; 2 Salvador BA Brasil Faculdade de Medicina, Universidade Federal da Bahia - Salvador (BA), Brasil.

**Keywords:** Alta do paciente, Estado funcional, Tempo de permanência, Hospitalização, Cuidados semi-intensivos, Atenção à saúde, Cuidados paliativos, Idoso, Unidades de terapia intensiva

## Abstract

**Objetivo:**

Descrever as trajetórias clínicas de pacientes que receberam alta diretamente de uma unidade de terapia intensiva para uma unidade de cuidados pós-agudos.

**Métodos:**

Trata-se de estudo de coorte retrospectivo de pacientes que foram transferidos de uma unidade de terapia intensiva ou unidade de cuidados intermediários para uma unidade de cuidados pós-agudos entre julho de 2017 e abril de 2023. O estado funcional foi determinado pelo escore da Medida de Independência Funcional.

**Resultados:**

Foram incluídos no estudo 847 pacientes, e a idade média foi de 71 anos. Foram admitidos 692 (82%) pacientes para reabilitação, enquanto 155 (18%) foram admitidos para cuidados paliativos. O tempo médio de internação na unidade de cuidados pós-agudos foi de 36 dias; 389 (45,9%) pacientes receberam alta para casa, 173 (20,4%) foram transferidos para um hospital de cuidados intensivos, e 285 (33,6%) morreram durante a internação, dos quais 263 (92%) tinham uma ordem de não ressuscitar. Dos pacientes admitidos para fins de reabilitação, 61 (9,4%) tiveram agravamento do estado funcional, 179 (27,6%) não tiveram alteração do estado funcional, e 469 (63%) tiveram melhora do estado funcional durante a hospitalização. Além disso, 234 (33,8%) pacientes tiveram suas metas de tratamento alteradas para cuidados paliativos - a maioria dos quais estava no grupo que não teve melhora no estado funcional. Os pacientes cujo estado funcional melhorou durante a hospitalização eram mais jovens, tinham menos comorbidades, menos hospitalizações anteriores, menores taxas de alimentação enteral e traqueostomia, pontuações mais altas na Medida de Independência Funcional na admissão à unidade de cuidados pós-agudos e maior probabilidade de receber alta para casa com assistência médica menos complexa.

**Conclusões:**

As unidades de cuidados pós-agudos podem desempenhar papel importante no cuidado dos pacientes após a alta das unidades de terapia intensiva, tanto para aqueles que recebem reabilitação, quanto cuidados paliativos, especialmente para aqueles com doenças mais graves que não podem receber alta diretamente para casa.

## INTRODUÇÃO

Sobreviventes de unidades de terapia intensiva (UTIs) podem ter comprometimento da qualidade de vida e desfechos desfavoráveis a longo prazo.^( 1 )^Por isso, há propostas de modelos de cuidados para pacientes com alta da UTI, como clínicas pós-UTI.^( 2 , 3 )^No entanto, alguns desses pacientes têm longas internações na UTI, com doenças mais graves e prolongadas, e não podem receber alta diretamente para casa após a estabilização clínica.^( 4 )^O modelo de assistência mais adequado para esses pacientes não está bem estabelecido,^( 5 )^mas o uso de unidades de cuidados pós-agudos (PACFs - *postacute care facilities* ) é um modelo alternativo, sendo que até 30% dos pacientes com doenças agudas nos Estados Unidos recebem alta de uma UTI para uma PACF.^( 6 )^Neste estudo, coletamos dados de pacientes que receberam alta diretamente de uma UTI ou unidade de cuidados intermediários (UCI) para uma PACF em Salvador (BA).

## MÉTODOS

Trata-se de um estudo de coorte retrospectivo aprovado pelo Comitê de Ética com dispensa do Termo de Consentimento Livre e Esclarecido (aprovação 53032821.0.0000.0047). Todos os pacientes que foram transferidos diretamente de uma UTI ou UCI para a PACF entre julho de 2017 e abril de 2023 foram incluídos no estudo. A PACF é uma instituição privada com 60 leitos com oferta ininterrupta de assistência médica e de enfermagem. A PACF permite que os pacientes sejam admitidos para reabilitação ou cuidados paliativos, incluindo pacientes que precisem de ventilação mecânica e terapia de substituição renal. Pacientes instáveis, como os que necessitam de medicamentos vasoativos, não estão aptos à admissão. Os serviços de reabilitação, incluindo fisioterapia, fonoaudiologia e terapia ocupacional são oferecidos diariamente. Pacientes encaminhados para reabilitação podem receber até 18 horas de terapia por semana na primeira semana de hospitalização. Outros profissionais da saúde, como assistentes sociais, nutricionistas, psicólogos e farmacêuticos clínicos, também fazem parte da equipe dessa unidade.

O estado funcional foi medido semanalmente pela Medida de Independência Funcional (MIF), com escores de 18 a 126 pontos. Os pacientes foram classificados como tendo dependência funcional total (MIF = 18), grave (MIF entre 19 e 60 pontos), moderada (MIF entre 61 e 103 pontos) ou leve/independência (MIF entre 104 e 126 pontos). Os pacientes também foram categorizados como apresentando piora no estado funcional, nenhuma mudança no estado funcional ou melhora no estado funcional, conforme a diferença entre a última medição e o escore da MIF de admissão.

As diferenças entre os grupos de pacientes foram avaliadas por Análise de Variância (Anova) ou teste do qui-quadrado para variáveis contínuas ou categóricas, respectivamente. A correção *post hoc* de Bonferroni avaliou diferenças entre cada par de categorias. Medidas repetidas dos escores da MIF foram avaliadas por testes *t* pareados ou testes de McNemar para variáveis contínuas ou categóricas, respectivamente. Valor de p < 0,05 foi considerado estatisticamente significativo. Todas as análises foram realizadas com o *Statistical Package for the Social Sciences* (SPSS) v 21.0.

## RESULTADOS

Durante o período do estudo, 2.022 pacientes de 17 hospitais diferentes foram admitidos na PACF, dos quais 847 (42%) foram admitidos diretamente da UTI ou UCI e incluídos no estudo. A maioria dos pacientes (692 [82%]) foi admitida para reabilitação, enquanto 155 (18%) foram admitidos para cuidados paliativos.

Os pacientes eram idosos, com média de idade ± desvio-padrão (DP) de 71 ± 17 anos, média ± DP de 2,6 ± 1,8 comorbidades e um tempo médio ± DP de internação no hospital de agudos de 35 ± 31 dias ( Tabela 1 ). Os motivos mais frequentes de internação hospitalar foram infecção por doença pelo coronavírus 2019 (COVID-19) (ou seja, pacientes com teste positivo para coronavírus da síndrome respiratória aguda grave 2 [SARS-CoV-2] e sintomas compatíveis com COVID-19), presente em 164 (25%) pacientes, e sepse, acidente vascular cerebral, neoplasias malignas e trauma ou cirurgia de urgência, presentes em 123 (18%), 116 (17%), 71 (11%) e 66 (1%) pacientes, respectivamente. Com relação à disposição de alta, 389 (45,9%) pacientes receberam alta para casa, 173 (20,4%) pacientes foram transferidos para um hospital de agudos e 285 (33,6%) pacientes morreram, sendo que para 263 (92%) desses últimos foi dada ordem de não ressuscitar. O tempo mediano (intervalo interquartil - IQ25% - 75%) de internação foi de 30 (12 - 52) dias para todos os pacientes e 45 (29 - 63), 16 (7 - 32) e 16 (7 - 35) dias para pacientes que receberam alta para casa, pacientes que morreram e pacientes que foram transferidos para um hospital de agudos, respectivamente.

**Tabela 1:** Características dos pacientes admitidos na unidade de cuidados semi-intensivos diretamente de uma unidade de terapia intensiva ou unidade de cuidados intermediários (n = 847), pacientes encaminhados para cuidados paliativos e reabilitação e pacientes encaminhados para reabilitação com diferentes trajetórias funcionais (n = 649)

Características		Todos os pacientes (n = 847)	Encaminhados para cuidados paliativos (n = 155)	Encaminhados para reabilitação (n = 692)	Valor de p *	Encaminhados para reabilitação com pelo menos 2 medições do escore da MIF durante a hospitalização na PACF (n = 649)			
						Piora do estado funcional durante a hospitalização (n = 61)	Sem alteração no estado funcional durante a hospitalização (n = 179)	Melhora do estado funcional durante a hospitalização (n = 409)	Valor de p †
Sexo masculino		441 (52,1)	76 (49)	365 (52,7)	0,403	27 (44,3)	99 (55,3)	215 (52,6)	0,329
Idade, média		71 ± 17	80 ± 15	69 ± 17	< 0,001	77 ± 14	71 ± 18	67 ± 17	< 0,001 ‡ § ¶
Comorbidades		2,6 ± 1,8	2,1 ± 1,9	2,6 ± 1,8	0,006	3,2 ± 1,8	2,6 ± 1,9	2,6 ± 1,8	0,042 §
Hospitalizações no ano anterior		0,86 ± 1,2	1,27 ± 1,48	0,76 ± 1,11	< 0,001	1,11 ± 1,26	0,81 ± 1,13	0,69 ± 1,05	0,017 §
Tempo de internação no hospital agudo (dias)		35 ± 31	24 ± 23	38 ± 32	< 0,001	36 ± 33	44 ± 38	36 ± 29	0,012 ¶
Unidade antes da transferência para a PACF					0,022				0,703
	UTI	543 (64,1	87 (56,1)	456 (65,9)		38 (62,3)	122 (68,2)	273 (66,7)	
	UCI	304 (35,9	68 (43,9)	236 (34,1)		23 (37,7)	57 (31,8)	136 (33,3)	
Alimentação enteral na admissão na PACF		567 (66,9	97 (62,6)	470 (67,9)	0,202	43 (70,5)	153 (85,5)	2487 (60,4)	< 0,001 ‡ ¶
Traqueostomia na admissão na PACF		288 (34,0	28 (18,1)	260 (37,6)	< 0,001	22 (36,1)	98 (54,7)	129 (31,5)	< 0,001 ‡ ¶
Ventilação mecânica na admissão na PACF		35 (4,1	8 (5,2)	27 (3,9)	0,476	3 (4,9)	9 (5)	13 (3,2)	0,508
MIF na admissão na PACF		3 5 ± 19	27 ± 20	37 ± 19	< 0,001	35,7 ± 15,9	26,4 ± 16,6	41,4 ± 19,2	< 0,001 ‡ ¶
Última MIF medida na hospitalização na PACF		48 ± 32	27 ± 20	51 ± 32	< 0,001	26,7 ± 13,5	26,4 ± 16,6	66,6 ± 31,1	< 0,001 § ¶
Categorias da MIF na admissão					< 0,001				< 0,001 ‡ ¶
	Dependência funcional total	191 (24,7)	59 (54,6)	132 (19,8)		0 (0)	99 (55,3)	31 (7,6)	
	Dependência funcional grave	501 (64,8)	42 (38,9)	459 (69,0)		55 (90,2)	73 (40,8)	319 (78,0)	
	Dependência funcional moderada	72 (9,3)	5 (4,6)	67 (10,1)		6 (9,8)	5 (2,8)	54 (13,2)	
	Dependência funcional leve/independência	9 (1,2)	2 (1,9)	7 (1,1)		0 (0)	2 (1,1)	5 (1,2)	
Categorias da MIF na última medição durante a hospitalização na PACF					< 0,001				< 0,001 § ¶
	Dependência funcional total	187 (24,5)	61 (59,2)	126 (19,1)		25 (41,0)	99 (55,3)	0 (0)	
	Dependência funcional grave	335 (43,9)	33 (32,0)	302 (45,8)		34 (55,7)	73 (40,8)	190 (46,5)	
	Dependência funcional moderada	168 (22,0)	7 (6,8)	161 (24,4)		2 (3,3)	5 (2,8)	150 (367)	
	Dependência funcional leve/independência	73 (9,6)	2 (1,9)	71 (10,8)		0 (0)	2 (1,1)	69 (16,9)	
Mudanças de metas de cuidados para cuidados paliativos		234 (27,6)	NA	234 (33,8)	NA	40 (65,6)	93 (52)	83 (20,3)	< 0,001 § ¶
Tempo de permanência na PACF		36 ± 34	22,6 ± 25	39,2 ± 35	< 0,001	41 ± 29	33 ± 51	44 ± 25	0,002 ¶
Disposição de alta					< 0,001				< 0,001 ‡ § ¶
	Morte	285 (33,6)	127 (81,9)	169 (24,4)		31 (50,8)	86 (48)	27 (6,6)	
	Transferência para hospital agudo	173 (20,4)	4 (2,6)	158 (22,8)		11 (18)	59 (33)	81 (19,8)	
	Alta para casa	389 (45,9)	24 (15,5)	365 (52,7)		19 (31,1)	34 (19)	301 (73,6)	
Morte com ordem de não ressuscitar entre os pacientes falecidos		263 (92,2)	127 (100)	136 (80,4)	< 0,001	30 (96,8)	71 (82,5)	21 (77,8)	< 0,001 ‡ §
Complexidade da assistência médica após a alta da PACF					0,196				< 0,001 § ¶
	Alta para casa sem assistência domiciliar	104 (12)	4 (3)	100 (14)		1 (2)	5 (3)	93 (23)	
	Alta domiciliar com assistência domiciliar de reabilitação	232 (27)	14 (9)	218 (32)		9 (15)	8 (4)	191 (47)	
	Alta domiciliar com assistência de enfermagem 24 horas	54 (6)	6 (4)	48 (7)		9 (15)	21 (12)	17 (4)	
Escore de satisfação com a hospitalização na PACF		9,5 ± 1	9,5 ± 1	9,5 ± 1	0,681	9,4 ± 0,9	9,5 ± 1,1	9,5 ± 1,1	0,718

MIF - Medida de Independência Funcional; PACF - unidade de cuidados pós-agudos; UTI - unidade de terapia intensiva; UCI - unidade de cuidados intermediários.

* Valor de p para comparação entre pacientes admitidos para cuidados paliativos ou reabilitação;

† valor de p para comparação entre pacientes com piora do estado funcional, sem alteração do estado funcional e com melhora do estado funcional durante a hospitalização;

‡ p < 0,05 entre piora do estado funcional durante a hospitalização *versus* nenhuma alteração do estado funcional durante a hospitalização;

§ p < 0,05 entre piora do estado funcional durante a hospitalização *versus* melhora do estado funcional durante a hospitalização;

¶ p < 0,05 entre sem alteração do estado funcional durante a hospitalização *versus* melhora do estado funcional durante a hospitalização. Resultados expressos como n (%) ou média ± desvio-padrão.

Os pacientes admitidos para cuidados paliativos eram mais velhos, tinham mais comorbidades, mais internações anteriores e apresentavam dependência funcional mais grave na admissão na PACF ( Tabela 1 ) do que os pacientes admitidos para reabilitação. No entanto, os pacientes que receberam cuidados paliativos tiveram um tempo de internação hospitalar menor e taxas mais baixas de traqueostomia. Os pacientes encaminhados para cuidados paliativos permaneceram na PACF por períodos mais curtos e tiveram maior probabilidade de morrer do que os pacientes encaminhados para reabilitação.

Dos pacientes admitidos para fins de reabilitação, 649 (93,8%) tiveram pelo menos duas medições do escore da MIF. Uma comparação do último escore da MIF com o escore da MIF de admissão revelou que 61 (9,4%) pacientes apresentaram piora no estado funcional, 179 (27,6%) não tiveram alteração no estado funcional e 469 (63%) apresentaram melhora no estado funcional durante a hospitalização. Os pacientes com melhora do estado funcional durante a hospitalização eram mais jovens, tinham menos comorbidades, menos hospitalizações anteriores, menores taxas de alimentação enteral e traqueostomia e pontuações mais altas da MIF na admissão na PACF ( Tabela 1 ). Os pacientes com melhor estado funcional tinham menor probabilidade de alteração de suas metas de tratamento para cuidados paliativos, menor tempo de internação na PACF e maior probabilidade de receber alta para casa com assistência médica menos complexa.

Com relação aos pacientes que foram admitidos para fins de reabilitação e receberam alta para casa e tiveram pelo menos duas medições do escore da MIF (n = 354), houve melhora na pontuação média da MIF, de uma pontuação média ± DP de 41,3 pontos ± 20,4 na admissão para uma pontuação média ± DP de 66,1 pontos ± 33,3 na alta (p < 0,001), com melhora no estado funcional de 301 (85%) pacientes durante a hospitalização. Essa melhora também foi observada na análise dos escores categorizados da MIF. Comparando os escores de admissão com os de alta, houve redução na proporção de pacientes com dependência funcional total de 37 (10%) para 23 (6%) e com dependência funcional grave de 263 (74%) para 142 (40%) e aumento na proporção de pacientes com dependência funcional moderada de 48 (14%) para 124 (35%) e de dependência funcional leve/independência de 6 (2%) para 65 (18%), p < 0,001, sendo que 175 (49,4%) pacientes melhoraram a categoria de estado funcional durante a hospitalização ( Figura 1 ).

**Figura 1:** Alterações nas categorias funcionais durante a hospitalização de pacientes admitidos para reabilitação, com pelo menos duas medições do escore da Medida de Independência Funcional, que receberam alta para casa, n = 354 (p < 0,001).

## DISCUSSÃO

Neste estudo, relatamos que os pacientes que foram transferidos de UTIs ou UCIs para PACFs eram pacientes idosos com multimorbidade, longas internações em UTIs e maior complexidade clínica. A maioria dos pacientes foi admitida para reabilitação, o que permitiu identificar três trajetórias diferentes de estado funcional, com variáveis associadas a cada trajetória. Além disso, observou-se com frequência a alteração das metas de cuidados para cuidados paliativos nessa população.

A gravidade da dependência funcional em nossa população de estudo indica um menor acesso a clínicas de seguimento convencionais, reforçando a necessidade de pesquisas específicas de modelos alternativos de cuidados.^( 5 , 7 )^No entanto, a maioria dos estudos em PACFs concentrou-se em uma ampla população de pacientes menos gravemente comprometidos. Por exemplo, pacientes incluídos em um estudo que avaliou uma intervenção de múltiplos componentes para transição em sepse eram mais jovens e tinham taxas mais baixas de admissão na UTI e um menor tempo de internação em hospitais de agudos.^( 8 , 9 )^

As taxas de retorno a hospitais de agudos foram comparáveis às encontradas na literatura^( 10 )^e não foram diferentes das taxas de reinternação de pacientes gerais pós-UTI e sépticos.^( 8 , 9 , 11 )^No entanto, a taxa de mortalidade geral foi maior do que a relatada anteriormente para pacientes gerais pós-UTI.^( 12 )^No entanto, a maior gravidade da dependência funcional nessa população indica a possibilidade de viés de seleção nas análises de mortalidade e reinternação, pois estudos anteriores geralmente acompanharam os resultados de longo prazo após a alta para casa, não incluindo mortes que ocorrem em PACFs ou que ocorreriam no hospital de agudos na ausência de transferência para uma PACF.

Com a avaliação das diferenças nas características dos pacientes transferidos para cuidados paliativos e para reabilitação, descobrimos que a maioria dos pacientes admitidos para reabilitação teve ganhos funcionais significativos. Além disso, conseguimos descrever três grupos diferentes de pacientes admitidos para reabilitação, com diferentes trajetórias funcionais durante a hospitalização, as quais foram associadas a características e desfechos clínicos. Esses achados podem ajudar a melhorar o prognóstico do paciente e alinhar as expectativas dos pacientes e de seus familiares.

Aproximadamente 30% dos pacientes tiveram suas metas de tratamento alteradas durante a hospitalização. Menos da metade dos pacientes costuma ter objetivos de tratamento formalmente alinhados de acordo com as suas preferências,^( 8 , 9 )^embora esse seja um dos elementos propostos de assistência após a UTI.^( 13 )^Não temos o momento exato da alteração das metas de assistência, portanto não foi possível verificar a direção da associação entre a alteração das metas de assistência e a trajetória clínica durante a hospitalização. No entanto, considerando o objetivo principal da reabilitação e a maior proporção de pacientes com alterações das metas de tratamento entre os pacientes cujo estado funcional não melhorou durante a hospitalização, levantamos a hipótese de que a alteração dos planos de tratamento foi influenciada pela falta de resposta às terapias fornecidas.

Trata-se de um estudo observacional e retrospectivo, portanto nossos achados devem ser considerados formadores de hipóteses. Além disso, apesar de incluir pacientes encaminhados de 17 hospitais diferentes, este foi um estudo de centro único com acesso limitado a dados de hospitalização aguda. No entanto, até onde sabemos, este é o primeiro estudo que aborda internações críticas em uma PACF no Brasil e nossos resultados podem ajudar a informar a pacientes, clínicos e formuladores de políticas sobre o uso de PACFs como um local alternativo de alta para pacientes graves.

## CONCLUSÃO

As unidades de cuidados pós-agudos podem desempenhar papel importante no tratamento de pacientes após a internação em uma unidade de terapia intensiva, especialmente para aqueles com doenças mais graves que não podem receber alta diretamente para casa.
